# Membrane Melatonin Receptors Activated Cell Signaling in Physiology and Disease

**DOI:** 10.3390/ijms23010471

**Published:** 2021-12-31

**Authors:** Georgi Nikolaev, Ralitsa Robeva, Rossitza Konakchieva

**Affiliations:** 1 Faculty of Biology, Sofia University “St. Kliment Ohridski”, 1504 Sofia, Bulgaria; r.konakchieva@biofac.uni-sofia.bg; 2 Department of Endocrinology, Faculty of Medicine, Medical University, 1431 Sofia, Bulgaria; rali_robeva@yahoo.com

**Keywords:** melatonin, G protein-coupled receptors, MAPK/ERK signaling, single-nucleotide polymorphisms (SNPs), diseases

## Abstract

The pineal hormone melatonin has attracted great scientific interest since its discovery in 1958. Despite the enormous number of basic and clinical studies the exact role of melatonin in respect to human physiology remains elusive. In humans, two high-affinity receptors for melatonin, MT1 and MT2, belonging to the family of G protein-coupled receptors (GPCRs) have been cloned and identified. The two receptor types activate Gi proteins and MT2 couples additionally to Gq proteins to modulate intracellular events. The individual effects of MT1 and MT2 receptor activation in a variety of cells are complemented by their ability to form homo- and heterodimers, the functional relevance of which is yet to be confirmed. Recently, several melatonin receptor genetic polymorphisms were discovered and implicated in pathology—for instance in type 2 diabetes, autoimmune disease, and cancer. The circadian patterns of melatonin secretion, its pleiotropic effects depending on cell type and condition, and the already demonstrated cross-talks of melatonin receptors with other signal transduction pathways further contribute to the perplexity of research on the role of the pineal hormone in humans. In this review we try to summarize the current knowledge on the membrane melatonin receptor activated cell signaling in physiology and pathology and their relevance to certain disease conditions including cancer.

## 1. Introduction

Melatonin (N-acetyl-5-methoxytryptamine) is a hormone secreted by the pineal gland, which plays important role in a wide array of physiological processes in mammals [1]. The functional activity of melatonin has a broad spectrum of action, with some of its functions still being studied. Melatonin and its metabolites have proved to be powerful antioxidants and published data show that this action may be attributed to receptor-independent function of the hormone [2]. Well documented findings on the biological significance of the hormone in mammals include control of retinal function, control of reproductive cycles in seasonally breeding animals and influence on the phases of 24-h behavioral rhythms (e.g., sleep-wake cycle), and participation in the control of thermoregulation and sleep. These functions are thought to be mediated by specific melatonin receptors. Their documented localization in the retina, anterior pituitary, and suprachiasmatic nuclei of the hypothalamus supports these claims [1]. Melatonin can be perceived as a signaling molecule with a unique 24-h profile of diurnal secretion—high during the respective night and low during the day. The characteristic circadian secretion is the result of the nocturnal synthesis of the hormone under the action of stimulatory signals. In vertebrates, these signals are generated by sympathetic nerve fibers, which deliver information from the suprachiasmatic nuclei (SCN), mainly through the release of the neurotransmitter norepinephrine [3]. For this reason, according to J. Arendt, the molecule is known as “the signal of darkness to body physiology” [4] with proven unique significance for the control of circadian and seasonal physiology, including reproductive behavior [5,6].

In humans, two different G-protein-coupled receptors which bind melatonin with high affinity have been identified and characterized [7,8]. They are thought to regulate a wide array of physiological activities in response to the rhythmic secretion of melatonin from the pineal gland. The most convincing data relate to research in animal models and indicate involvement of the melatonin-dependent pathways in the control of circadian rhythms, retinal physiology, memory, immunomodulation, and tumorigenesis [9,10].

The action of melatonin is also probably mediated by activation of the ROR/RZR group of nuclear receptors [11]. The subfamily of receptors that, according to some authors, bind melatonin include: RZRα, RORα, RORα2 and RZRβ. The structure of nuclear receptors consists of an N-terminal domain, a DNA binding domain that contains a zinc double finger domain, a hinge region, and a ligand-binding site at the carboxyl terminus. RZRβ receptors are found in nerve tissue and RZRα in adipose tissue, skin, testes, cartilage, and liver [12].

Although melatonin receptors have already been cloned in a number of species and their distribution and characteristics extensively studied [8,12,13,14,15], evidence of hormone-receptor-activated signaling events in particular cell targets are few and the meaning of their physiological relevance remains questionable. Also, little is known about the physiological relevance of the different types of melatonin receptors. A major drawback in studies focused on melatonin receptor signaling is the inability to identify a physiological stimulus associated with the activation of melatonin receptor-driven intracellular transduction.

## 2. Melatonin Receptors

### 2.1. Melatonin Receptor Subtypes—Classification and Physiological Significance

The first pharmacological characterization of a functional mammalian melatonin receptor [16] and the cloning of the first human melatonin receptor [13] came 25 and 36 years, respectively, after melatonin itself was discovered [17]. Around 2000, a family of melatonin receptors associated with G proteins was cloned in various mammalian species, including fish, amphibians, birds, mice, sheep, and humans.

The receptor was found to be expressed at high levels in the suprachiasmatic nuclei of the hypothalamus, which helped to explain the inverse modulating effect of the indolamine on physiological rhythms [18]. Following the introduction of 2-(125I) iodomelatonin as an isotopic indicator for autoradiography and radioreceptor analysis, specific binding sites have been found in several brain areas [19], as well as in some peripheral organs [20].

Melatonin receptors are named and classified according to structural and operational criteria defined by the International Union of Pharmacology (IUPHAR) Committee on Receptor Nomenclature and Drug Classification. The operational criteria are fulfilled by a pharmacological profile of specific ligands at the receptor recognition site usually from agonist/efficacy and antagonist dissociation constants obtained in native tissues. Receptors are classified according to evidence of a particular transduction mechanism and demonstration of endogenously expressed receptors. Melatonin receptors were named for their endogenous ligand melatonin, which is abbreviated as “MT” using capital letters, and each type of receptor was denoted by a numerical subscript (i.e., MT1, MT2). According to the “Concise Guide to PHARMACOLOGY 2015/16” [21] produced in conjunction with NC-IUPHAR to provide the official IUPHAR classification and nomenclature for human drug targets, melatonin receptors are included in the rhodopsin family (Class A) of G protein-coupled receptors and are activated by the endogenous ligands melatonin and N-acetylserotonin, as well as by clinically used drugs like ramelteon, agomelatine and tasimelteon [22]. MT1/MT2 heterodimers present different pharmacological profiles from MT1 and MT2 receptors.

The MT3 melatonin binding site, also termed the ML2 receptor was studied predominantly in hamster brain and peripheral tissues such as kidney and testis. It was identified as a homologue of the human quinone reductase-2 enzyme [8,23].

Another distinct receptor which belongs to the Gi/o family of G proteins is expressed in Xenopus melanophores and chick brains. It was suggested that GPR50 is the mammalian counterpart of it although there is no evidence that melatonin can bind to GPR50 receptors [24]. GPR50 is regarded as an orphan receptor which is structurally related to the melatonin receptors with no melatonin binding capacity, hence termed melatonin-associated receptor. Data are reported that it can heterodimerize with either of the melatonin receptors in a constitutive manner [25,26]. The heterodimerization of GPR50 with MT1 abolishes the agonist binding and G protein-coupling capacity of the MT1 protomer, whereas it does not alter that of MT2 in the MT2/GPR50 heterodimer [25].

The human genes which encode the proteins MT1 and MT2 melatonin receptor are termed *MTNR1A* and *MTNR1B* respectively. *MTNR1A* and *MTNR1B* show distinct chromosomal localization. *MTNR1A* is localized to human chromosome 4q35.1 By contrast, *MTNR1B* maps to human chromosome 11q21–22. The proteins are composed of 350 and 365 amino acids and their predicted mass is 39,374 and 40,188 Da respectively. However, these numbers do not take into account possible post-translational modifications. The amino acid homology for the human MT1 and MT2 melatonin receptors is approximately 60% overall and 73% within the transmembrane domains. The human MT1 receptor shows more similarities with the rodent MT1 receptors than with that of bovine, ovine, and porcine MT2 receptors.

### 2.2. Melatonin Receptors MT1 and MT2 Belong to a Distinct Family of GPCR

The MT1 and MT2 melatonin receptors comprise their own subgroup within the GPCR superfamily [9,21,27]. They possess similar pharmacological characteristics and show high affinity for melatonin at subnanomolar concentrations [15]. Upon activation, both MT1 and MT2 regulate similar signaling pathways primarily via Gi/o proteins and β-arrestin1/2 [9,27,28], although they can also signal through Gq/11 and G16 proteins depending on the cellular type and tissue context [29,30].

Both melatonin receptors have a general structural motif consisting of seven transmembrane (TM)-spanning α-helical segments connected by alternating intracellular and extracellular loops, with the amino terminus located on the extracellular side and the carboxyl terminus on the intracellular side These seven α-helical segments contain stretches of 20 to 25 predominantly hydrophobic residues that span the cell membrane. In the family of rhodopsin/β2-adrenergic receptor, sequence homology between melatonin receptors and other G-protein-coupled receptors is observed in the transmembrane domains [8]. The amino terminus of MT1 has two consecutive N-terminal asparagine-linked glycosylation sites, while MT2 has only one site [8]. The carboxyl terminus of both receptors has consensus sequences for casein kinase 1α, casein kinase II, and protein kinase C, as well as postsynaptic density 95/disc-large/zona occludens binding domains that may be involved in membrane localization and traffic. What distinguishes the melatonin receptor family from other GPCRs is that they have an NRY motif downstream of the third transmembrane domain and NAXIY in transmembrane motif 7, instead of DRY and NPXXY motifs, respectively [8].

The MT1 receptor is associated with Gi and in particular Giα2, Giα3, and Gq/11. MT1 receptors are expressed in the brain, cardiovascular system (including peripheral vessels, aorta and heart), immune system, testes, ovaries, skin, liver, kidneys, adrenal cortex, pancreas, placenta, breast, retina, pancreas and spleen. Central action on circadian rhythms, sleep and thermoregulation most likely involves target sites in the hypothalamus, cerebellum, hippocampus, substantia nigra, and ventral tegument [12].

Activation of MT2 also leads to activation of Gi. The MT2 receptor is found in the immune system, brain (hypothalamus, suprachiasmatic nucleus), retina, pituitary gland, blood vessels, testicles, kidneys, gastrointestinal tract, mammary glands, adipose tissue, and skin [12].

Despite the high sequence similarity, differences in the intrinsic signaling capacity of MT1 and MT2 have been documented: MT2 has been shown to inhibit cGMP production [31] while MT1 can activate Gs in some experimental models [32].

### 2.3. MT1 and MT2 Functional Capacity Depends on the Formation of Homo- and Heterodimers

***MT1 receptor***. The computer model used to show the putative structure of the melatonin receptor binding site is based on rhodopsin. This model is analyzed by site-specific mutagenesis. Studies have shown that the binding site is similar to those of other G-protein-coupled receptors in the rhodopsin/β2-adrenergic receptor family. For example, His195 in the putative TM5 is conserved at all melatonin receptors and the position is identical to that used at the ligand-binding sites of other rhodopsin-like GPCRs. The model indicates that the histidine residue may form a hydrogen bond with an oxygen atom of the 5-methioxy group of melatonin. The model also suggests that Val192, which is located around a helical bend of His195 facing the hydrophobic binding pocket, is important for binding to the methyl portion of the methoxy group of melatonin. Thus, the computer model shows one of the sites playing a role in melatonin binding, but others have yet to be identified [8].

A second method for determining important residues for receptor binding and activation is the modification of conserved AK residues in rhodopsin-like GPCR [8]. The protonation of aspartate/glutamate in the conserved D/ERY motif on the cytoplasmic side of TM3 is thought to be involved in receptor activation. Ligand binding leads to deprotonation of these amino acids, which in turn leads to activation/proven at the rhodopsin receptor [33]. Changing the D/E to a neutral amino acid to mimic the exposed state results in constitutive activation and improved pairing of many rhodopsin-like receptors. The melatonin receptor is unique in that it has an NRY motif instead of a D/ERY motif. The change of NRY to ARY—imitation of the deprotonated active state, reduces the binding to such an extent that it is impossible to measure receptor activation. The change of the NRY motif in the melatonin receptor to the D/ERY motif reduces the binding affinity and the capacity of melatonin to activate the receptor. Therefore, unlike other rhodopsin-like G-protein coupled receptors, the melatonin receptor does not require deprotonation of the NRY motif to be activated, and Asn is required for optimal ligand binding and activation [8].

Melatonin receptors have two conserved cysteines (Cys127, Cys130), between helix III and the second intracellular loop, a region that is important for binding the receptor to the G-protein. The replacement with serine in the MT1 receptor indicates that they are required for normal G-protein activation and receptor trafficking. TM 3,5,6,7—mainly TM3 and TM7, are considered to be of particular importance for ligand binding in rhodopsin. The TM3 domains of Class A G-protein coupled receptors have a large number of Ser/Thr/Cys residues. They form hydrogen bonds with the peptide backbone, and therefore bend and twist the helices. Therefore, different hydrogen bonds will lead to different TM3 conformations, which represent the different functional states of the receptor-ligand bound or not, activated or not [34]. As with other G-protein-coupled receptors, the residues in the TM2 of MT1 are important for ligand binding [8]. Replacing Ser110 and Ser114 but not Ser103 in alanine reduces melatonin binding [35]. By inducing point mutations in the transmembrane domains of MT1, scientists have shown that some specific AKs in TM6 play an important role in binding. Replacing glycine with threonine (G258T) reduces receptor binding and activation. Mutations in glycine 258 are particularly important for maintaining the structure of MT1, as AK is thought to be positioned against the hydrophilic receptor nucleus [8].

***MT2 receptor.*** Melatonin receptors, like most other G protein-coupled receptors, have conserved cysteine in extracellular loop 1 and loop 2. Mutation in these cysteine residues in rhodopsin, δ-opioid, platelet-activating factor and M3 muscarinic receptors demonstrates the critical function of the disulfide bond for proper receptor conformation for ligand binding, receptor activation and surface activation [36]. However, these conservative cysteines are not always involved in the formation of disulfide bonds (e.g., the β2-adrenergic receptor) [37]. The disulfide bond formed between Cys113 and Cys 190 has been shown to play an important role in maintaining the correct conformation of the MT2 receptor so that ligand binding can occur without altering its expression on the cell surface [38]. Whether this disulfide bond is formed at a single melatonin receptor or between two dimer-forming receptors is not clear [8].

Key conservative AKs are involved in the ligand binding of MT2 receptors. This has been identified in binding studies following alanine mutation. Mutation in Asn175 at TM4 or His208 at TM5 at the MT2 receptor significantly reduced the binding affinity for melatonin. Asn175 in TM4 appears to aid in the binding of the 5-methoxy group of the melatonin molecule to the receptor. His208 in TM5 is critical for receptor binding [39]. Mutation in Ser123 or Ser127 in TM3 or Ser293 in TM7 of the MT2 receptor has no effect on binding affinity, while equivalent serine (Ser110 and Ser114 in TM3) are considered critical for the binding of melatonin to hMT1. Therefore, the binding sites of the two receptors have similar histidine residues (His195/208 in TM5), but also different residues required for ligand binding. Several AKs in TM V (Val204), VI (Leu272), and VII (Tyr298) have been identified that are involved in MT2 binding site interactions. Pro174, Pro212, and Pro266 have been shown to be particularly important for receptor binding and/or signaling. The identification of these specific motifs may lead to the design of specific therapeutic compounds [8].

Using bovine rhodopsin as a source. a model was created of the binding sites of MT1 and MT2, based on mutagenesis analysis and 3D-homologous modeling of the receptor. In these models, the binding site of the MT1 receptor appears to be smaller than that of the MT2 receptor. Conservative histidines of TM5 (His195 for MT1 and His208 for MT2), which are thought to bind to the methoxy group, are found at both binding sites [8].

Specific features in the molecular structure of MT1 and MT2 receptors have been identified and this suggests the presence of potentially different ligand recognition binding sites [39,40]. The capacity of MT1 and MT2 receptors to form homo- and heterodimers was initially demonstrated in HEK293 cells [41] and later the pharmacological profile of MT1/MT2 heterodimers was shown to be different than that of MT2 homodimers [42]. The data about melatonin receptor oligomerization was further expanded by the discovery that both MT1 and MT2 form heterodimers with the orphan receptor GPR50 and that a loss of receptor function in the case of MT1/GPR50 dimers can occur [43]. The report that MT1/MT2 heterodimers preferentially activate the Gq/PLC/Ca^2+^ pathway [44] is among the few shedding light on the physiological sense of the melatonin receptors’ oligomers.

### 2.4. MT Receptors Are Widely Distributed in Central and Peripheral Tissue

As shown in other species [45], in humans MT receptors are widely present in central nervous system and in various peripheral organs.

***MT1 receptor***

In the human brain, the MT1 receptor is found mainly in the hypothalamus, cerebellum, hippocampus, the substance *Nigra*, and the ventral tegmental region [10]. Peripheral distribution includes cardiovascular system (including peripheral blood vessels, aorta, and heart), the immune system including spleen and lymph nodes, testes, ovaries, skin, liver, kidneys, adrenal cortex, placenta, mammary gland, and the pancreas [10,15,46,47].

***MT2 receptor***

The distribution of MT2 receptor is more limited than that of the MT1 receptor, but its expression has been found in many tissues and organs, including the immune system and the reproductive system [47], the brain hypothalamus/suprachiasmatic nucleus (SCN), the retina, the pituitary gland, blood vessels, reproductive cells, and tissue [47,48], testes, kidneys, gastrointestinal tract, mammary gland, adipose tissue and skin [15,49,50].

### 2.5. MT1 and MT2 Receptor Sensitivity Is Regulated by GPCR Common Mechanisms and Circadian Fluctuation of Melatonin Concentration

Regulation of signal transduction is essential for maintaining a timely and efficient cellular response and homeostasis. Activation of G-protein-coupled receptors according to milieu and physiology context leads alterations in receptor sensitivity—desensitization, increased sensitivity, receptor import, and trafficking, which contribute to ligand-dependent efficacy of transduction [51]. MT1 and MT2 receptors are typically and differently regulated by physiological (30–400 pM) and supraphysiological (1–1000 nM) melatonin concentrations. Physiological concentrations of melatonin at night (100–400 pM) are above the effective ligand concentration for melatonin receptors, which are activated by picomolar concentrations of melatonin [52,53]. The day concentrations are usually below 30 pM and yet they induce activation and desensitization of melatonin receptors after prolonged exposure to the hormone (about 8 h). Blood levels of melatonin after taking an oral dose of 0.3 mg are similar to endogenous levels at night in humans [54]. Oral doses of melatonin and other ligands of 1 mg or more may increase its level in the blood several times above the concentration required to activate the melatonin receptor and may therefore alter receptor sensitivity [54,55].

An exposure period comparable to a normal night’s duration—about 8 h, does not influence receptor density, affinity and functional sensitivity as observed inMT1 expressed by heterologous mammalian cells in mammals. However, in suprachiasmatic cultures in vitro the density of receptors increases, and their affinity decreases [56].

Recovery of MT2 receptors after melatonin-mediated desensitization/internalization depends in part on new protein synthesis. Re-sensitization of MT2 receptors after exposure to physiological levels of melatonin lasts up to 8 h, while exposure to supraphysiological levels leads to stronger desensitization and a longer recovery period—up to 24 h, to reach baseline levels. The impact of circadian melatonin production on processes of desensitization and/or internalization of MT1 and MT2 receptors may underlie the melatonin receptor—dependent feedback in the suprachiasmatic nucleus to fine tune circadian rhythms driven by the master clock [8].

The phenomenon seems not confined only to SCN. Melatonin was shown to increase the differentiation of human adult mesenchymal stem cells into osteoblasts by activating MT2 receptors. A reduction in the MT2-mediated decrease in alkaline phosphatase enzyme activity is observed when the MT2 receptors are completely desensitized, suggesting that a decrease in receptor sensitivity is a necessary step in the above differentiation [57].

These findings suggest that human MT1 and MT2 receptors can be desensitized under the influence of circulating melatonin in a manner similar to that observed in animals. A strong pattern of a 24-h rhythm in melatonin receptor expression has been reported in the *Pars tuberalis* in various species [58,59]. For many tissues, the presence of a pronounced circadian rhythm in the biological response to the action of melatonin has been shown, as well as changes in the biochemical characteristics of the binding receptors [60]. In an autoradiography study using 2-(125I) iodomelatonin to detect melatonin receptors in rat secondary lymphoid organs isolated at regular intervals on a 24-h basis, we were able to register marked diurnal variations in the capacity of the specific binding sites [61]. Maximum specific binding was found 1 h before the onset of the dark phase and the start of rise in evening endogenous melatonin measured in plasma, which was indicative of the presence of sensitivity to the action of the hormone in secondary immune organs. Evidence from similar research demonstrate that this phenomenon is at least partly driven by melatonin-induced receptor autoregulation [40,58,59,62]. It is conceivable that the receptor sensitivity to the ligand is regulated by the exposure duration to physiological or supra-physiological peripheral melatonin levels [8]. This is particularly important in view of the role of the hormone as a photoperiod chemical transducer to body physiology and hence intimate cellular function. The significance of circadian synchronization of main endocrine axes for the operational functioning of proper signaling mechanisms and physiological adaptation has been well documented in humans [63].

## 3. MT1 and MT2 -Activated Signaling Pathways Are Involved in Physiological Processes

**Figure 1:**
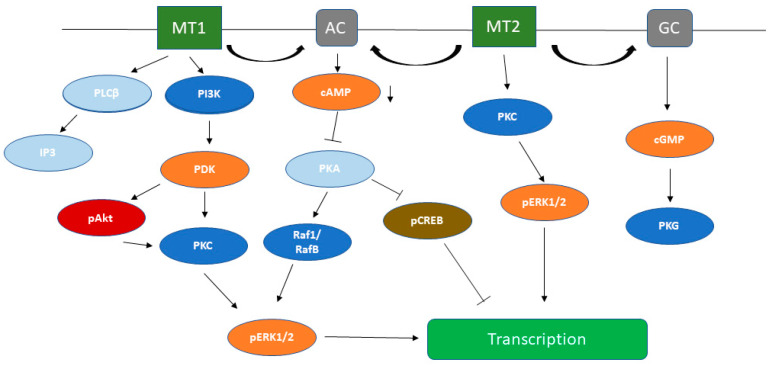
Melatonin receptor-activated signaling pathways. AC—adenylate cyclase; GC—guanylate cyclase; PLCβ—phospholipase C-β; IP3—inositol triphosphate; PI3K—phosphoinositide 3-kinase; PDK—phosphoinositide-dependent kinase; PKC -protein-kinase C; pAkt—phosphorylated protein-kinase B; cAMP—cyclic adenosine monophosphate; PKA—protein-kinase A; pCREB—phosphorylated cAMP response element binding protein; cGMP—cyclic guanosine monophosphate; PKG—protein-kinase G; pERK1/2—phosphorylated extracellular signal-regulated protein kinases 1 and 2.

As members of the GPCRs family, melatonin receptors may act on a variety of cellular signaling pathways through heterotrimeric GDP/GTP-linked proteins. Activation of the receptors causes dissociation of the heterotrimeric G-proteins and the resulting Gα subunit and Gβγ complex interact with various effector molecules involved in cellular signaling (Figure 1). Among the effector systems that are activated by melatonin are adenylate cyclase, phospholipase C, phospholipase A2, potassium channels, guanylyl cyclase, calcium channels [9,28,64].

### 3.1. cAMP Signaling Pathway

Upon activation of MT1 and MT2 receptors, AC is initially inhibited by PTX (pertussis toxin)—sensitive Gi proteins (Gαi2 and Gαi3 isoforms) [30]. The decrease in cAMP subsequently inhibits the activity of protein kinase A and the phosphorylation of nuclear factor CREB (cAMP responsive element binding protein). Melatonin has a role in the regulation of the rhythmic expression of the so-called “clock genes” through AC/cAMP signaling [28]. Phosphorylated CREB can bind to the promoters of these genes and increase their expression [65]. The melatonin-induced signaling cascade in SCN can modulate circadian rhythms by counteracting the effect of PACAP (pituitary adenylyl cyclase activating peptide), which induces CREB phosphorylation [66].

### 3.2. PLC Signaling Pathway

Upon binding of MT1 to Gq proteins or via Gi—Gβγ dimers, melatonin stimulates PLC activity, which converts phosphatidylinositol (PIP2) to diacylglycerol (DAG) and inositol 1,4,5-triphosphate (IP3). Elevated levels of secondary mediators activate protein kinase C (PKC), activate calcium signaling by calmodulin kinases (CaMK), and also stimulate mitogen-activated protein kinases (MAPKs), including ERK, JNK, and p38.

### 3.3. Melatonin-Induced Activation of MEK/ERK Kinases

Reports linking the activation of melatonin receptors to alterations in the extracellular signal-regulated (ERK) kinases signaling pathway [67] have opened a new venue for research of its effects on cell metabolism, cell cycle and gene expression. As an endogenous physiological regulator of the rest-activity cycle melatonin may be intimately involved in the control of cell growth, proliferation, and differentiation, at least in part through the control on gene expression by the ERK pathway.

In addition to the inhibition of CREB, activation of ERK1/2 MAP kinases in lamb pinealocyte cultures by melatonin receptors was shown to be facilitated through stimulation of Gi/o proteins [68]. Furthermore, Chen et al. demonstrated that ERK1/2 activation by MT1 is only mediated though Gi/o proteins, while MT2 is dependent on the cooperative activation of Gi/o and Gq/11 proteins. In the absence of Gq/11 proteins, however, MT2-induced ERK1/2 activation switches to a β-arrestin1/2-dependent mode. The signaling cascade downstream of G proteins is the same for both receptors and involves activation of the PI3K/PKCζ/c-Raf/MEK/ERK cascade [69]. This same work suggests that plasticity of ERK activation by MT2 could be explained by the participation of the receptor in MT1/MT2 heterodimers, which reveals a new mechanism underlying tissue-specific responses to melatonin.

Recent research from our laboratory demonstrated that melatonin treatment stimulates the expression of pERK1/2 in human foreskin fibroblasts [70]. The data suggest that the activation of MT1 melatonin receptor is probably related to phosphorylation of ERK1/2 at least in expanding fibroblasts, which subsequently may act to alter gene expression and regulate cell fate. The case was not the same in granulosa cell cultures where melatonin showed no effect on the activation of ERK1/2. Similar results of Wang et al. show that steroidogenesis in theca cells is stimulated by melatonin via activation of the PI3/AKT pathway by MT1 and MT2, but not ERK1/2 [71]. Involvement of the same signaling pathway in answer to melatonin treatment promotes neural differentiation of pluripotent stem cells [72]. However, this was not the case in embryonic stem cells, where prolonged exposure to melatonin was found to contribute to maintenance of their pluripotency state in a MT1-dependent manner [73].

A recent study also demonstrates the capability of melatonin to stimulate the regeneration of motor neurons. This pro-regenerative action is again MT1-mediated, and at least in part due to a sustained activation of the ERK1/2 pathway [74]. On the contrary, the activation of melatonin MT1 and MT2 receptors in ELT3 leiomyoma cells by melatonin reduced cell proliferation and downregulates Akt-ERK1/2-NFκB signaling pathway [75]. These authors report melatonin-induced apoptosis and autophagy cell death progression in ELT3 cells.

## 4. Do MT Signaling Matters to Human Reproductive Function?

Studies in the last forty years have established unequivocally that the seasonal reproductive cycle in animals is under melatonin regulation. However, the exact sites of melatonin action in the reproductive system are not clear. Theoretically, site(s) of melatonin action on the reproductive system can be the hypothalamus, pituitary, gonads, male and female reproductive tract, male accessory sex organs, mammary gland or any combination of the above targets, which has led some authors to the hypothesis of multiple sites for melatonin action on mammalian reproductive system [76].

Considerable evidence shows that melatonin plays important role in many aspects of the natural reproductive performance in mammals. The hormone has been shown to have a direct effect on the ovarian function and reproductive cycles, to have potential role in the pathophysiology of various reproductive problems such as endometriosis, polycystic ovary syndrome (PCOS) and premature ovarian failure (POF).

It is generally accepted that the regulatory effect of melatonin in the reproductive system is realized through its action in the hypothalamus and the anterior pituitary gland [77]. However, the presence of melatonin receptors has been demonstrated in granulosa cells of ovarian follicles and, in addition, higher concentrations of melatonin have been found in human preovulatory follicular fluid than in plasma. These data are indicative of the direct involvement of the hormone in ovarian processes [78,79], in addition to its regulatory effect in the hypothalamic-pituitary-ovarian axis. In the ovary, melatonin has a direct effect on steroidogenesis in granulosa cells and folliculogenesis [78,79,80]. The expression of MT1 and MT2 receptors has been established in many tissues and organs, including the reproductive system. Melatonin binding receptors are present in the membrane fraction of human granulosa cells [79]. MT1 and MT2 in human granulosa-luteal cells were identified by RT-PCR [78,81].

Granulosa-lutein cells (GLC) isolated from donors of preovulatory follicular fluid during assisted reproduction procedures represent an interesting model to study cellular plasticity and intercellular communications. The proliferation and differentiation of GLCs are of critical importance for the regulation of follicular growth, ovulation and luteinization process in the ovary. The presence of melatonin receptors (MR) has been demonstrated in ovarian granulosa cells [81] and higher MEL level compared to the concentration in peripheral blood has been reported in preovulatory follicular fluids [82]. These data suggest potential direct action of melatonin in ovarian function [78,83] in addition to its regulatory role at the level of the hypothalamus-pituitary-ovarian axis. Furthermore, the presence of melatonin and its precursors—serotonin and N-acetylserotonin—has also been documented in human ovarian extracts in addition to key enzymes involved in the melatonin synthesis [84]. There are data that the pineal hormone stimulates the production of progesterone and androstenedione [85] without effect on estrogen and mRNA level for the estrogen-converting enzyme aromatase (P450arom) [86]. In contrast, we were able to show stimulation of P450arom protein expression in GLC cultures by in vitro melatonin at nanomolar concentration corresponding to levels of the hormone found in follicular fluid. At first glance this is somehow contradictory to previous reports showing inhibitory melatonin effect on P450arom in breast cancer cells [87]. Considering that the ovary-specific promoter of the P450arom gene (*CYP19A1*) has a binding motif for cAMP and the pituitary follicle-stimulating hormone (FSH) exerts transcriptional control on the gene by a cAMP-dependent mechanism, it is conceivable that melatonin may also influence the expression of the P450arom enzyme responsible for the conversion of androgens to estrogens by granulosa cells. The role of melatonin in follicular steroidogenesis is complex and may vary depending on the cell type (follicular/stromal), mode of treatment, species, and dose [78,84]. Moreover, melatonin has been shown to stimulate progesterone production by granulosa cells, but to exert no effect on estrogen levels [85,86]. One possible explanation might be linked with other aspects of granulosa cell function in growing follicles. Recent reports demonstrated stimulation by melatonin of the beta-catenin anti-apoptotic pathway and mitochondrial function [88], which seems to be important for the FSH/cAMP stimulation of aromatase in granulosa cells during follicular differentiation [89]. One important function of melatonin present in follicular fluid seems to be related to the regulation of the androgen-converting enzyme and hence the differentiation status and competence of granulosa cells serving the growing oocyte. This view was supported by the observation of an enhancement of the signal for alpha-tubulin under melatonin treatment as well as of colocalization of MT1 and alpha-tubulin in the perinuclear area where the Golgi apparatus and microtubules interact to organize vesicular and organelle trafficking [90].

Another intriguing aspect of melatonin action as circadian message is its possible interaction with cytoskeleton and interference with cell cycle control and progression [91,92]. Chronic melatonin exposure of Chinese hamster ovary cells results in MT1 receptor desensitization and depolymerization of microtubules, most probably via activation of PKC by melatonin receptors and further phosphorylation of calmodulin and activation of Ca^2+^/CaM kinases [93]. Studies on microtubule polymerization have shown that melatonin effects on cytoskeleton at nanomolar concentration are mediated by its antagonism to Ca^2+^/calmodulin [91]. The mode of melatonin antagonistic action includes prevention of the inhibition of microtubule assembly by competing for Ca^2+^ thus causing microtubule enlargement. Most probably the signaling cascade involves modulation of melatonin receptors shown to interact with the microtubule polymerization and depolymerization processes [92,93,94]. Importantly, MT1 and MT2 immunofluorescence signals were upregulated under the treatment with melatonin in human GLC cultures and associated cell differentiation effects suggested important role of the pineal hormone in ovarian follicle development, which remains to be elucidated.

The expression and functional meaning of melatonin receptors on human spermatozoa emerged as an interesting avenue of research along with the development of in vitro fertilization procedures and the need to achieve high-quality and healthy gametes. Based on numerous reports demonstrating antioxidant activity of the pineal hormone [23,95] studies exploiting melatonin usage envisage improvement of individual assisted reproduction approaches, contingent on motility characteristics and viability of gametes during in vitro fertilization (IVF) procedures. Unfortunately, in many cases, extremely high in vitro concentrations of melatonin have been reported, which could never be reached under physiological conditions of circadian timed release of the pineal hormone, thus raising the question of the physiological relevance of the induced cellular response [96,97].

Activation of MT1 membrane receptors by physiological doses of melatonin is coupled to inhibition of cAMP accumulation in the target cells [8], which makes research on membrane receptor-mediated signaling in spermatozoa very attractive. Cyclic AMP is very important for gametes function because sperm flagellar movement and capacitation occur in a cAMP-dependent manner and melatonin effect on sperm motility has been documented in studies including humans, but data are controversial [98].

Single reports of melatonin concentration in human semen indicate very low picomolar levels which seem reasonable for the activation of putative membrane melatonin receptors on human spermatozoa [99]. In earlier studies, melatonin specific binding with receptor characteristics has been shown in mammalian spermatozoa by in vitro ligand-binding assays [100]. Using purified ejaculated spermatozoa from healthy donors we were able to demonstrate the expression of MTRN1A melatonin receptor gene and corresponding protein distribution in human spermatozoa [48]. In pilot experiments to compare MT1 and MT2 distribution in healthy controls and infertile men MT2 shows lower intensity of the signal which in general overlaps with the MT1 signal but extends along the whole tail early after purification (data not published). Relative quantification by means of real-time RT-PCR revealed gene expression level regardless of the presence of seminal plasma comparable to that found previously [101]. The result eliminated the possibility of an inhibitory effect of semen plasma on the MTNR1A gene expression while masking effect on the receptor protein could not be excluded. The confocal immunofluorescence signal for MT1 receptor protein showed characteristic localization in the neck and mid-piece of spermatozoa, which supports the view of membrane receptor-mediated role of melatonin on sperm cellular movements. Nevertheless, functional responses and transduction pathways linked to MT activation have to be further elucidated.

## 5. What Is behind the Neuroimmunomodulatory Action of Melatonin

In addition to its role as a circadian regulator of physiological functions, the pineal hormone melatonin exerts cell-protective activity through involvement in processes such as proliferation and differentiation, antioxidant protection, apoptosis, and mitochondrial homeostasis. It has been shown that the hormone is able to influence both the innate and adaptive immune response by its effect on cell proliferation of immunocompetent cells, cytokine secretion [102] and to increase the weight of immune organs under normal and immunosuppressive conditions [103]. For a number of tissues, the presence of a pronounced circadian rhythm in the biological response to the action of melatonin has been shown, as well as changes in the biochemical characteristics of binding receptors [104]. The abundance and demonstrated function of melatonin receptor is summarized in Table 1. Most likely, the specific response of the immune system to melatonin action is closely dependent on the level and circadian changes in the expression of MT receptors. Using different in-vivo and in vitro approaches to investigate adaptive physiological responses in laboratory rodents during chronic glucocorticoid exposure we have demonstrated that melatonin plays an important role in neuroimmune adaptation and is able to stimulate the in-vivo stress-protective immune response by the presence of high affinity specific membrane receptors [20].

In vitro experiments have shown the presence of specific melatonin receptors in human cultures of T-mitogen-activated lymphoid cells with a dissociation constant Kd = 140–230 pM [47]. The functionality of the identified receptor on mitogen-stimulated lymphocytes is confirmed by the activation of the receptor by physiological doses of exogenous melatonin sufficient for the manifestation of antiproliferative effect in cultures, as well as by the dose-dependent nature of the demonstrated inhibitory effect. The single class of specific high-affinity binding sites for 2-[125I] iodomelatonin, with a pharmacological profile and Kd in the range of 100–200 pM is close to those found for the membrane-bound receptor in other organs and species [104] and falls within the affinity range reported for human hypothalamus, kidney and granulosa cells [79,118,119], as well as human MT1 and MT2 receptors in transfected cell lines [67,120].

In our studies to investigate MT1 and MT2 in resting and PHA-stimulated peripheral blood mononuclear cells /PBMC/ we routinely use suprananomolar concentrations of melatonin to activate endogenous high affinity receptors. Employing quantitative cell-based image technology [121] we were able to demonstrate modulation of cell cycle phase distribution of PBMC [48] in contrast to other studies employing high doses of melatonin [122,123]. Along with restriction of cell cycle entry arrest at G0/G1, the progression to S phase was delayed in stimulated cells treated with melatonin and polyploidy (DNA content > 4 n) was significantly enhanced without changes in pre-phase cells. We were able to show by confocal immune fluorescence that human quiescent and stimulated PBMC express MT1 and MT2 receptors, implicating direct responsiveness to melatonin stimulation by the dose used. Our results substantiate findings by other authors who reported expression of both receptor types in the thymus, spleen, and lymphocytes [20,103,124] proposed role for MT2 in inhibition of leukocyte rolling [102] and reduction of chemotaxis towards CXC chemokines [125]. Notably the results supported the specific and different pattern of distribution of both MT1 and MT2 receptors described to be present in humans [8,15,29]. Since both receptors are supposed to be activated by the dose used, it is difficult to interpret their respective role in activation changes linked to TCR expression, migration, and differentiation (assessed by estimation of CD25+) [43]. Nevertheless, the observed expansion of membrane bound signal, associated with extensive intercellular contacts points out at the involvement in the control of cell spreading and immune sensing. The signal for MT1 in resting PBMC resembles a cap structure known to describe TCR in silent T-lymphocytes [126,127]. Also very importantly, melatonin was able to cause rearrangement of peripheral to perinuclear translocation of actin microfilaments early upon activation along with significant reduction of the nuclear area index. An interesting future research would be to investigate the role of melatonin to the formation of immunological synapses, known to go along with cell cycle changes [128,129,130].

Recent research on TREGs modulation by melatonin demonstrated that the hormone exerts immunoregulatory activity in myasthenia gravis (MG) by balancing effector and regulatory Th cell populations as well as by suppressing pro-inflammatory cytokine production [131]. Serum melatonin levels were found lower in MG patients than in healthy controls, and MT1 expression was lower in PBMCs from MG patients than in those from controls. Administration of melatonin significantly decreased Th1 and Th17 cell responses and proinflammatory cytokine production. Further investigation in vitro revealed that melatonin administration increased FoxP3 and IL-10 expression in CD4+ T cells from MG patients and enhanced the suppressive function of Tregs.

## 6. MT—Dependent Signaling of Cell Growth—An Important Clue for the Oncostatic Activity of Melatonin

Low levels of melatonin in the body in old age correlate with an increased risk of developing malignancies, which is consistent with the notion that cancer is a disease of old age. Prolonged exposure to artificial blue light until late in the day can lead to disturbances in melatonin synthesis, which also increases the risk of developing various cancers, including breast, colorectal, liver and lung cancers [132].

MT1 has been found in multiple sites in the human body, but also under conditions of cancer of the prostate, breast, bone, gallbladder, melanoma, Warthin’s tumor [133]. The biological role and clinical relevance of the MT1 in normal and tumor tissues are poorly understood. MT1 was found to modulate the proliferation of malignant cells and was reported to mediate the effects of melatonin on growth suppression and gene modulation in breast cancer cells [134]. The tumor cells exhibit intense expression of the cytokeratins associated with columnar differentiation and it has been reported that activation of MT1 increases phosphorylation of mitogen-activated protein kinase and MEK1-2 and ERK 1/2, probably leading to induction of synthesis of filamentous structures of non-neuronal tissues [27]. Authors hence hypothesize that the MT1 may actively participate in synthesizing cytokeratin in Warthin’s tumor cells.

The antitumor effects of melatonin are related to its ability to inhibit the proliferation, angiogenesis, and migration of cancer cells and to stimulate their apoptosis, alone or by potentiating the antiproliferative, anti-invasive and proapoptotic effects of various chemotherapeutic agents. In breast cancer cells these effects are MT1-mediated and include inhibition of phosphorylation of Akt, ERK and PKC [135,136]. Inhibitory effects were also reported for ovarian cancer where the pineal hormone inhibits Akt, p38 MAPK and mTOR signaling [137].

Studies performed with the A549 adenocarcinoma cell line show decreased expression of proinvasive factors, such as MLCK (myosin light-chain kinase), with concomitant positive regulation of components and regulators of tight junctions, such as occludin and osteopontin, which aid in the targeting of occludin-containing vesicles. These effects are thought to be mediated at least in part by melatonin-mediated inhibition of the JNK/MAPK signaling pathway, which significantly reduces the migration potential of treated cells [138]. Regarding apoptosis, melatonin was found to increase the antitumor activity of berberine in two adenocarcinoma cell lines (H1299 and A549) by inhibiting AP-2β/hTERT, NF-κB/COX-2 and Akt/ERK signaling pathways and activating cytochrome C and the mitochondrial apoptotic pathway [139]. This study also shows that melatonin alone can stimulate the activation of ERK (to a greater extent) and that of Akt (to a lesser extent) in H1299. Most likely, the induction of the ERK pathway is due to activation of PKC in the presence of melatonin. Calmodulin, which is a substrate of both above kinases (PKC and ERK), after phosphorylation can be localized in the area of the formed stress fibers, where it can stimulate their construction, stabilization and contractility by activating CaMKII/Pyk2/RhoA and MLCK [140,141,142]. This could be one of the possible explanations of the potential of melatonin to modulate cancer cell invasion and metastasis. Regarding focal adhesive contacts, it has also been found that in MCF-7 physiological concentrations of melatonin can switch the migration profile of cells to stationary by increasing the number of these contacts and the stress fibers mediated by ROCK activation [143].

## 7. Melatonin Receptors and Melatonin Receptor Polymorphisms—Clinical Impact

### 7.1. Diabetes Mellitus, Melatonin and Melatonin Receptors

#### 7.1.1. Melatonin and Diabetes Mellitus

Diabetes mellitus (DM) represents a complex heterogeneous group of disturbances characterized by hyperglycemia, including type 1 DM (T1DM), type 2 DM (T2DM), gestational DM (GDM) and other specific types of DM [144,145]. The proper regulation of insulin secretion and insulin sensitivity is crucial for the maintaining of glucose homeostasis. Thus, the autoimmune impairment of β-cell induces the development of type 1 DM (T1DM), while the pronounced insulin resistance in the context of inadequate compensatory insulin secretory response due to β-cell dysfunction leads to type 2 DM [144,145,146]. The deterioration of insulin sensitivity is the primary process leading to stimulation of β-cells and hyperinsulinemia. The development of islet cells dysfunction and diminished insulin secretion over time might provoke a continuum of carbohydrate disturbances including increasing fasting and postprandial glucose levels and subsequent development of T2DM [147]. However, many other factors such as adipocyte insulin resistance, decreased incretin effect, increased glucagon concentration, dysfunction of renal proximal tubular glucose reabsorption as well as impaired brain regulation of appetite and thermoregulation might promote the raise of blood glucose levels in susceptible individuals [148].

Interestingly, an additional factor contributing to the “ominous octet” of T2DM pathophysiology might be the disturbed circadian rhythm, which is a common finding in shift workers and in individuals exposed to light pollution at night-time [149]. Night shift work has been associated with increased risk of obesity, metabolic disturbances and T2DM [150,151]. Moreover, it might deteriorate the glycemic control in patients with T1DM and T2DM [152,153]. Simulated night work in volunteers have been associated with progressive decrease of melatonin production [154]. The melatonin secretion alteration during night work might reflect the increased exposure to light, and effects of sleep deprivation, but it is also an important marker of the circadian rhythm disruption [154,155]. These findings raise the question if the decrease in melatonin secretion is associated with the increased risk of carbohydrate alterations and T2DM. However, the answer is not unambiguous so far.

The acute administration of low dose melatonin impairs the glucose tolerance and insulin sensitivity in postmenopausal women [156]. Similarly, Rubio-Sastre et al. have found that the acute administration of melatonin in high dose in women deteriorates the glucose tolerance regardless of the time of the day. The morning increase of glucose levels after acute melatonin administration has been related to decreased insulin secretion, while the evening increase has been associated with pronounced insulin resistance [157]. The acute supraphysiological melatonin treatment in healthy men has also been related to increased insulin resistance [158]. However, the chronic administration of melatonin for 10 weeks has not been associated with significant changes of metabolic parameters in patients with metabolic syndrome [159], while night-time melatonin treatment might even improve glycemic control in diabetic patients [160]. Recently, a large meta-analysis has confirmed the beneficial influence of melatonin supplementation on fasting glucose levels, insulin resistance and glycated hemoglobin in diabetic patients at both lower and higher doses [161].

Consequently, the melatonin effects on carbohydrate homeostasis might depend on different factors including duration of administration, age, health status and concomitant disorders of the patients as well as endogenous melatonin levels. A strong correlation between melatonin and insulin secretion during the night has been described in young patients with metabolic syndrome, but not in healthy controls [162]. A large study based on the Nurses’ Health Study cohort has found an independent association between melatonin secretion estimated by urinary 6-sulfatoxymelatonin levels and the subsequent development of type 2 diabetes. Individuals in the lowest category of melatonin secretion have shown more than doubled risk of T2DM in comparison to participants in the highest category [163]. Moreover, melatonin could influence the glycemic control of patients with T2DM [164]. Probably, optimal melatonin levels are needed for intact carbohydrate metabolism and both supraphysiological and low endogenous melatonin levels might have detrimental effects on glucose metabolism. The “equilibrium hypothesis” has suggested that both amplification and reduction of melatonin signaling could induce carbohydrate disturbances [165]. Another interesting hypothesis assumes a functional antagonism between melatonin secretion and food intake [166]. Low melatonin levels during the day potentiate normal glycemic tolerance after food load, while high melatonin levels during night fasting ensure pancreatic beta cell recovery [166]. In case of increased melatonin concentrations during periods of eating pathological changes in glucose metabolism emerge as in night-eaters and shift-workers [166]. Despite the contradictory findings and hypotheses, it is now clear that the pineal hormone exerts fine tuning of the carbohydrate metabolism through its receptors on pancreas, liver, and adipose tissue [162]. Recent studies focused on melatonin receptors and their polymorphisms have gathered new data about the important link between melatonin signaling and development of carbohydrate disturbances.

#### 7.1.2. Melatonin Receptors, Pancreas and Diabetes Mellitus

Experimental studies have revealed different melatonin signaling pathways influencing insulin secretion. Melatonin downregulates adenylate cyclase/cyclic adenosine monophosphate pathway through MT1 and MT2 receptors as well as the guanylate cyclase/cyclic guanosine monophosphate pathway mediated by MT2 receptors thus inhibiting insulin secretion of the beta cells. On the other side, melatonin could also stimulate insulin secretion by inositol trisphosphate-release mediated by MT2, but inhibitory signals are predominant [165,167]. Despite the convincing evidence in animal studies the data about melatonin receptor expression in human pancreatic cells are rather contradictory. MT1 and MT2 receptor expression have been shown in Langerhans islets of human pancreatic tissue with MT1 being predominantly expressed. Interestingly, strongly increased mRNA expression levels of both MT1 and MT2 receptors have been found in samples from diabetic patients in comparison to those of non-diabetic donors [167]. Other studies have also proved both MT1 and MT2 receptor expression in beta-cells of human pancreatic tissue [168,169]. In islet samples from both diabetic and non-diabetic donors the lowest relative MT1- and MT2-receptor density was observed in alpha-cells, whereas the highest density of both receptors was measured for beta-cells in non-diabetic and delta-cells in diabetic islets [168]. On the opposite, Ramracheya et al. have observed predominantly MT1 and very low MT2 mRNA expression in the alpha-cells of human islets, but not in the beta cells. According to them melatonin stimulates glucagon secretion from alpha-cells with a subsequent paracrine-mediated indirect increase of insulin levels from beta-cells [170]. Interestingly, melatonin also modulates the secretion of somatostatin from the delta-cells of both non-diabetic and diabetic donors [168].

**Figure 2:**
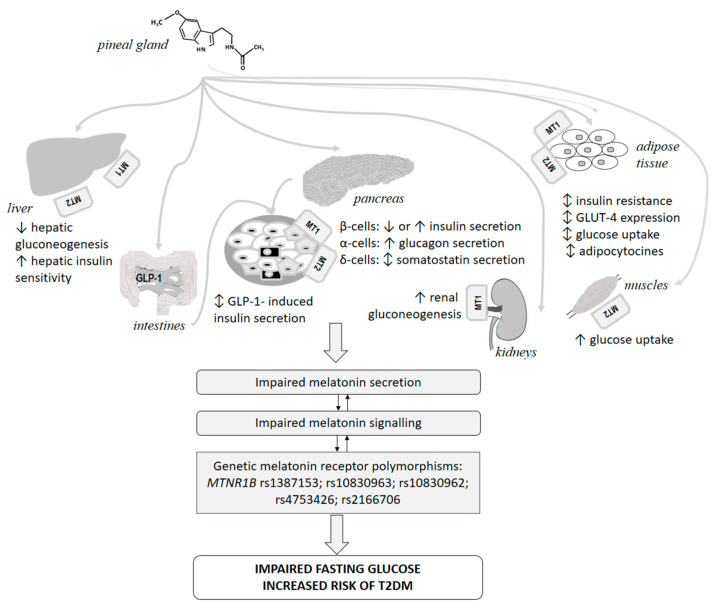
Melatonin and carbohydrate disturbances—pathophysiological mechanisms. T2DM—diabetes mellitus type 2; GLP-1—glucagon like peptide 1; MT1—melatonin receptor type 1; MT2—melatonin receptor type 2; ↑—increase; ↓—decrease; ↕—modulate.

Despite the important influence of melatonin signaling on insulin, somatostatin and glucagon secretion, the pineal hormone dysregulation might be involved also in other pathophysiological pathways related to the development of T2DM. Animal studies have shown that melatonin could modulate the insulin response to glucagon-like peptide 1 [171], renal tubular gluconeogenesis [172], hepatic insulin sensitivity [173] as well as hypothalamic-liver regulatory mechanisms [174]. Further studies in humans are needed to reveal the precise role of melatonin and its signaling pathways in the complex pathophysiology of T2DM (Figure 2).

#### 7.1.3. Melatonin Receptor Polymorphisms and Glucose Abnormalities

The identification of *MTNR1B* gene encoding the MT2 receptor protein as an important diabetic gene associated with pancreatic beta-cell dysfunction a decade ago gives a huge impetus to the research focused on associations between pineal hormone signaling and receptor polymorphisms on one hand and carbohydrate abnormalities on the other hand [169,175,176,177]. The *MTNR1B* rs10830963 C>G variant has been associated with increased fasting glucose in a large cohort of European descent. Moreover, the presence of the minor G allele of the same polymorphism has been related to an increased T2DM risk in a meta-analysis of 18,236 cases and 64,453 controls (OR 1.09/1.05–1.12/) [177]. The rs10830963 G allele determines not only increased fasting glucose levels but also a lower early insulin response to glucose load [169,178]. Moreover, individuals carrying the rs10830963 G-allele have shown higher expression of MT2 receptor in the beta-cells in comparison to carriers of the C allele, especially after the age of 45 years [169]. However, the rs10830963 polymorphism has not been associated with impaired glucose tolerance or peripheral insulin resistance, though it influences the hepatic insulin resistance in elderly patients [178]. Since 2009 a myriad of studies has confirmed the negative influence of the rs10830963 polymorphism on fasting plasma glucose and T2DM risk, though ethnic differences have also been described (Table 2). According to several meta-analyses the *MTNR1B* rs10830963 polymorphism determines increased risk of T2DM, with the strongest associations found in Caucasians and South Asians [177,179,180]. Interestingly, the longitudinal follow up of a large cohort of individuals has shown that the *MTNR1B* polymorphic variant increases the rate of progression from normal glucose levels to impaired fasting glucose levels, but not the transition from impaired fasting glucose state to overt T2DM [181]. Despite the logical assumption, the rs10830963 variant does not mediate the well-known associations between sleep disturbances, depressive symptoms and T2DM [182,183].

Additionally, several meta-analyses have proved an association between the *MTNR1B* rs10830963 variant and the development of gestational diabetes mellitus (GDM) [269,270,271,272]. The GG genotype carriers have shown 78% increased risk of GDM development in comparison to CC carriers irrespective of ethnicity [270]. Moreover, the effect of lifestyle intervention on the development of GDM has been significantly blunted in women carrying the rs10830963 G allele in comparison to C allele carriers [234].

A strong association has been described also between the *MTNR1B* rs10830963 polymorphic variant and the presence of a latent autoimmune diabetes in adults with low GAD autoantibodies [245]. Thus, the presence of the rs10830963 polymorphic G allele might predispose to different carbohydrate disturbances in adult individuals.

Currently, there are only a few studies investigating the functional relationship between endogenous melatonin circadian rhythm, exogenous melatonin administration and the presence of the *MTNR1B* rs10830963 polymorphism. Lane et al. have shown that the presence of the rs10830963 risk G allele causes a later melatonin offset and prolonged melatonin production extending later into the morning irrespective of sleep duration [273]. Moreover, the increased T2DM risk associated with the rs10830963 variant might be modulated by sleep timing with later wakening being protective [273]. A large UK study has shown a close relationship between T2DM risk and the late chronotype of the patients. However, in individuals with rs10830963 GG genotype the evening chronotype does not increase the risk for carbohydrate disturbances [274]. Nevertheless, late evening dinner promotes the development of glucose disturbances in GG genotype carriers [275]. Additionally, the high-dose melatonin administration in the morning or in the evening has also been associated with rs10830963 polymorphism-dependent changes in the glucose levels and insulin secretion [276,277]. Probably, the late sleep timing and avoidance of food in the early morning and in the late evening might be important preventive strategies for the rs10830963 G allele carriers at increased T2DM risk, while the attempt to change their chronotype are not justified [273,274,277]. The gathering of additional information about circadian rhythms, melatonin signaling pathways, as well as carbohydrate disturbances and their complications considering genetic melatonin receptor polymorphisms is of huge clinical importance.

Recently, Tan and Benedict have shown 19% increased risk of fatal or non-fatal myocardial infarction in diabetic patients carrying the rs10830963 G allele [278]. Prediction of complications in diabetic patients based on their genetic background might be an important step towards personalized medicine and health improvement.

Other *MTNR1B* receptor polymorphisms have also been investigated in regard to carbohydrate abnormalities, though not in details. The *MTNR1B* rs1387153 C>T variant modulates the risk for T2DM and GDM in different ethnic groups. The minor T allele has been associated with increased fasting glucose levels, reduced beta-cell function and higher prevalence of T2DM in European, but not in Asian populations [188,192,279,280]. Nevertheless, the rs1387153 CC genotype has been shown to be protective in regard to T2DM development in Chinese individuals [281]. Additionally, rs1387153 polymorphism has been associated with increased risk of GDM in Caucasian, Middle Eastern and Asian populations [237,238,272]. Both polymorphisms rs1387153 and rs10830963 are in strong linkage disequilibrium at least in some ethnic groups, so that their effects on the glucose abnormalities might be dependent of each other [188].

Other *MTNR1B* polymorphisms have also been studied in the context of carbohydrate metabolism. Two *MTNR1B* SNPs rs10830962 C>G and rs4753426 T>C determine higher fasting plasma glucose levels and lower insulin secretion even after adjustment for multiple confounders, while the *MTNR1B* SNP rs12804291 C>T does not influence glucose or insulin concentrations [176]. Interestingly, the *MTNR1B* SNP rs3781638 A>C minor allele C has been associated with lower fasting glucose concentrations, optimal insulin release and improved insulin sensitivity in the same study [176]. Several studies have confirmed the associations between rs4753426 polymorphism and GDM, while the impact of rs10830962 for the GDM onset is still not clarified [228,229,240,272,282]. The SNP rs2166706 T>C near *MTNR1B* has also been associated with increased glucose levels, reduced pancreatic β-cell function, and higher risk of T2DM and GDM [185,240].

Apparently, common *MTNR 1B* receptor polymorphisms leading to amplification of melatonin receptor signaling exert unfavorable effects on carbohydrate metabolism and increase the risk of T2DM development with approximately 10%. However, the very rare *MTNR1B* variants determining partial or total loss of receptor function are associated with much more magnified risk of T2DM [283]. At present, these contradictory results cannot be explained, despite the multiple suggested hypotheses.

In opposite to *MTNR1B* genetic polymorphisms, *MTNR1A* genetic variants are rarely investigated in regard to glucose abnormalities, despite the widespread distribution of MT1 receptor in pancreatic islet tissues. The common *MTNR1A* rs2119882 T>C variant has not been associated with the GDM risk in Polish pregnant women but increased the risk of GDM in Chinese individuals [229,235]. More studies are needed to reveal the pathophysiological associations between different genetic variants associated with the melatonin receptor signaling and metabolic disturbances.

### 7.2. Oncological Diseases, Melatonin and Melatonin Receptors

#### 7.2.1. Melatonin, Melatonin Receptor Polymorphisms and Breast Cancer

The melatonin might exert oncostatic effects on hormone-dependent and hormone-independent tumors [284,285,286,287]. Most investigations are focused on melatonin secretion and signaling in breast cancer. Melatonin might suppress breast cancer growth through its antioxidant, immunomodulating, antiestrogenic, anti-angiogenic, anti-proliferative and proapoptotic effects with many of these effects being mediated through MT1 receptor [136,288,289,290]. The latter inhibits estrogen response genes and estrogen receptor-α transcriptional activity, decreases aromatase activity and modulates growth factors and proto-oncogenes in breast tumor cells [136,291,292,293,294]. The heterodimerization of MT1 and MT2 receptors might enhance their signal transduction, thus both MT1 and MT2 might have important influence on cancer suppression as shown in different animal and human studies [41,136].

The ageing in female rats is associated with a significant decrease of night melatonin secretion and diminished MT1 receptor expression. Lower endogenous melatonin levels and decreased receptor sensitivity provoke mammary tumor growth in older animals [295]. Similarly, lower levels of melatonin have been found in breast cancer patients when compared to healthy females irrespective of sleep quality or light pollution [296]. Moreover, higher night 6-sulfatoxymelatonin levels predict 19–22% lower risk of breast cancer after six to twelve years of follow-up in the Nurses’ Health Study cohort irrespective of MT1 receptor status [297,298]. These results have been supported by a meta-analysis including five prospective case-control studies that proves an inverse association between the melatonin metabolite concentrations and breast cancer risk [299]. However, a subsequent larger meta-analysis does not corroborate the same interrelation, and the higher melatonin secretion tends to be protective only for postmenopausal women (RR  =  0.88, [0.75–1.02], *p*  =  0.10), and patients with estrogen-receptor-positive tumors (RR  =  0.83, [0.64–1.07], *p*  =  0.15) [300]. Nevertheless, the increased MT1 receptor expression has been related to slower disease progression and better overall survival not only in tamoxifen-treated patients with hormone-responsive tumors but also in women with triple-negative breast cancer [301,302]. Therefore, melatonin supplementation has been considered as a promising adjuvant therapeutic option for prevention and treatment of breast cancer [294,303].

Despite the important associations between the MT1 and possibly MT2 receptors and the breast cancer risk, melatonin receptor polymorphisms are rarely investigated in patients with breast tumors. According to a large Korean study the *MTNR1A* rs2119882 T>C C allele carriers show significantly decreased breast cancer risk in comparison to other women [304]. Additionally, *MTNR1A* rs13113549 variant has not been associated with increased breast cancer risk in a large cohort of Norwegian nurses, but its recessive homozygous genotype exerts protective influence on cancer risk in individuals with three consecutive night shift per month [263]. Deming et al. have investigated eight *MTNR1A* (rs6838290; rs2165667; rs6847693; rs2165666; rs7665392; rs12642043; rs4861722; rs10030173) and four *MTNR1B* (rs4611171; rs10765576; rs10830962; rs10830963) polymorphisms regarding breast cancer. The results show that only one of the investigated *MTNR1A* polymorphisms might modulate the breast cancer risk. The *MTNR1A* rs7665392 T>G minor allele is protective for postmenopausal women but increases the cancer risk in premenopausal women [262]. The authors assume that *MTNR1A* rs7665392 variant might be related to decreased expression or effects of MT1, but functional studies are actually lacking.

Deming et al. have not established any associations between *MTNR1B* rs4611171, rs10830962, and rs10830963 polymorphisms and breast cancer risk [262]. The same *MTNR1B* rs10830963 variant has not been associated with increased tumor risk in the aforementioned cohort of Norwegian nurses, however the *MTNR1B* rs10830963 GG genotype doubled the risk of breast cancer in nurses exposed to four or more consecutive night shift per month in comparison to participants rarely exposed to night work [263]. The *MTNR1B* rs10765576 A>G polymorphism has also been investigated in women with breast tumor with contradictory results [262,305].

Apparently, the impact of *MTNR1A* and *MTNR1B* receptor polymorphisms on breast cancer development and progression might be modulated by distinct endogenous and exogenous factors, e.g., ethnic differences, age, menopausal status, individual chronotype, exposure to night shift-work and light pollution. Further studies on melatonin signaling might help to identify women at increased risk of malignancy who might benefit from melatonin administration or work-schedule changes. The potential of melatonin-tamoxifen drug conjugates as novel anticancer drugs also needs to be clarified in future studies [306].

#### 7.2.2. Melatonin, Melatonin Receptor Polymorphisms and Other Malignancies

Melatonin might suppress the development and progression of different reproductive system tumors in men and women, e.g., prostate and ovarian malignancies. Melatonin inhibits the proliferation of prostate cancer cells through MT1 receptor-mediated mechanisms including activation of protein kinase A and protein kinase C, inhibition of the NF-κB activation and modulation of androgen receptor signaling [307,308]. Moreover, melatonin might impair the interrelations between the NF-κB and androgen receptor splice variant 7, thus detaining the development of resistance to androgen depletion therapy in advanced prostate cancer [309]. The oncostatic effects of melatonin include also induction of apoptosis, modulation of prostate tumor metabolism, decreased angiogenesis, and regulation of neuroendocrine differentiation, suppression of sirtuin-1 and local growth factors as well as amplification of cytokine-induced toxicity [290,310].

Animal and human studies have proved the beneficial effects of melatonin in prostate cancer. Melatonin administration in TRAMP (Transgenic Adenocarcinoma of Mouse Prostate) mice reduces the prostatic volume and increases the degree of differentiation of cancer cells. Moreover, the hormone decreases Ki-67 and sirtuin-1 expression as well as local production of insulin-like growth factor 1 [311]. In men, low night melatonin secretion has been associated with increased risk of advanced prostate carcinoma [312]. Lissoni et al. has shown that administration of the melatonin might have clinical benefits for metastatic prostatic cancer patients resistant to treatment with gonadotropin releasing hormone analogues [313]. Recently, a large Russian study showed the effect of long-lasting adjuvant melatonin administration in a dosage of 3 mg taken 30 min before sleep in prostate cancer patients with different prognosis. The patients had undergone standard combined hormonal and radiation therapy and were followed up to 19 years. The overall survival rate of patients with favorable and intermediate prognoses was very good and did not depend on melatonin treatment. However, in the group with advanced disease, the median overall survival of patients taking melatonin was twice as high as that of non-treated individuals (153.5 months vs. 64.0 months, *p* < 0.0001) [314].

Despite the important associations between the pineal hormone, MT1 receptor and prostate cancer, studies investigating the *MTNR1A* genetic polymorphisms in regard to the prostatic diseases are lacking. Only one study aiming to reveal common genetic risk factors for T2DM and prostate cancer has investigated the *MTNR1B* rs10830963 polymorphism. The variant has been nominally related to 10% increased risk of prostate cancer, but without statistical significance after adjustment for multiple comparisons [261].

Melatonin could suppress ovarian cancer cells through direct intracellular actions as well as MT1-mediated signaling pathways [315]. Melatonin administration reduces the growth of ovarian masses and the development of adenocarcinomas in laboratory animals [316]. Additionally, melatonin limits endometrial hyperplasia in rats exposed to high-dose estradiol and suppresses succinate-mediated growth of endometrial carcinoma cells with MT1 being a significant enhancer of the inhibitory effect [317,318]. Accordingly, the MT1 and MT2 receptor inactivation precludes the pronounced growth-suppressing effects of the melatonin on leiomyosarcoma tissues [319]. The pineal indolamine exerts also proapoptotic and antioxidative effects on cervical cancer cells and has shown promising results as an adjuvant therapeutic agent to conventional chemotherapy regimens [320,321]. Nevertheless, clinical studies focused on melatonin treatment in female reproductive cancer patients are lacking. Additionally, melatonin receptor polymorphisms have not been studied in women with reproductive system tumors apart from breast cancer.

The melatonin signaling could be important factor influencing the development and progression of many non-reproductive tumors such as gastrointestinal, liver, pancreatic, lung, renal, oral, skin, bone and haematological malignancies [290,322]. In almost all of them melatonin could favor apoptosis and inhibit growth, angiogenesis, and the dissemination of metastases through direct or receptor-mediated mechanisms. Additionally, it might exert anti-oxidant and immune-modulatory effects, and could be used as an adjuvant therapy reducing side effects and enhancing the potency of chemo- and radiotherapy [290,322].

Considering the important associations between melatonin-receptor mediated signaling and solid tumors it is interesting to know if the melatonin receptor polymorphisms might modulate the malignancy risk in different populations. However, specific conclusions could not be drawn, since only a few studies on the topic mostly in Asian populations have been published. In a large Taiwan study, five *MTNR1A* SNPs (rs13140012, rs6553010, rs2119882, rs13113549, and rs2375801) as well as five *MTNR1B* SNPs (rs1387153, rs1562444, rs4611171, rs10765576, and rs10830963) were evaluated in patients with hepatocellular carcinoma cancer (HCC). The carriers of *MTNR1A* rs6553010 GG genotype showed 58% higher risk of HCC development in comparison to A allele carriers after adjustment for age and alcohol use. Other investigated melatonin receptor polymorphisms did not increase separately the risk of HCC onset, but specific *MTNR1A* and *MTNR1B* haplotypes were associated with increased HCC risk. Interestingly, the presence of *MTNR1A* rs2119882 or rs2375801 minor alleles increased the risk of developing distant metastasis five to sevenfold. The authors suggested that rs2119882 polymorphism might alter the MT1 regulation and expression, thus decreasing receptor-mediated anti-metastatic effects [264]. Additionally, a study searching for common genetic risk factors for T2DM and pancreas cancer in a cohort of European ancestry has found slightly increased nominal risk for pancreatic cancer in *MTNR1B* rs10830963 polymorphism carriers, without convincing evidence for pleiotropic effects of the *MTNR1B* variant [265]. Another Taiwanese population study has investigated the interrelations between oral cavity cancer and *MTNR1A* polymorphisms. No significant differences between *MTNR1A* rs2119882, rs13140012, and rs6553010 variant carriers and wild-type individuals have been established in regard to oral cancer prevalence. However, the presence of combined *MTNR1A* rs2119882 T>C, rs13140012 A>T, and rs6553010 A>G CTA haplotype has been related to 77% increased risk of oral malignancy in comparison to the common TAA haplotype. The *MTNR1A* polymorphic variants show important synergistic influence on the oral cancer development in patients with underlying risk factors for the disease such as betel quid chewing and smoking [323]. These results are in accordance with the fundamental research of Nakamura et al. showing that the lack of MT1 receptor expression might predict increased tumor growth and worse prognosis in patients with oral squamous-cell cancer [324]. The *MTNR1A* rs2119882 T>C, rs13140012 A>T, and rs6553010 A>G polymorphisms have been investigated also in patients with urothelial carcinoma and healthy controls without significant differences in genotype distribution. However, the presence of rs6553010 minor G allele in homozygous or heterozygous condition has been associated with significantly increased risk of invasive carcinoma in comparison to AA genotype [325]. Consequently, the *MTNR1A* polymorphisms might play important role for the development and clinical progression of different hormone-independent cancers, and they could promote carcinogenic effects of different exogenous factors. On the opposite, the influence of *MTNR1B* variants on carcinogenesis is still unrevealed.

## 8. Conclusions

The functional activity of melatonin has a broad spectrum of action, with some of its receptor-mediated effects still being not well understood. By activating G-protein bound receptors in various target tissues, melatonin regulates circadian rhythms, brain, vascular, metabolic, reproductive, endocrine, and immune functions. MT1 and MT2 regulated expression in a wide range of cells in the periphery, including lymphocytes, fibroblasts, granulosa, sperm, etc. suggests that melatonin may interact with nuclear and cytoskeletal structures, possibly affecting various cellular functions, such as cell cycle control, subcellular organization, and genomic stability most importantly in a circadian rhythmic fashion.

The G-protein bound melatonin receptor—dependent signaling might have important role for the clinical practice as a modulator of immune, metabolic, and reproductive processes as well as oncogenesis. Moreover, the melatonin receptor genetic polymorphisms might determine different susceptibility to pathological conditions and complications provoked by internal or external stressors. The MT1 and MT2 receptor expression in pancreatic islets could influence the insulin, glucagon and somatostatin secretion. Some common *MTNR1B* receptor genetic polymorphisms modulate the fasting glucose levels and early insulin secretion from beta-cells, thus influencing the T2DM and GDM risk in different ethnic groups. Moreover, the same polymorphisms could determine different effects of lifestyle interventions and/or medical treatment in case of metabolic disorder. The most investigated *MTNR1B* rs10830963 variant has been associated with a prolonged melatonin production that could facilitate the development of hyperglycemia under specific environmental conditions. The gathering of additional information about circadian rhythms, endogenous melatonin signaling pathways, and melatonin receptor polymorphisms in patients with carbohydrate disturbances could help for the development of new preventive strategies based on individual circadian rhythm and genetic features.

Recent findings have shown the important potential of melatonin as an oncostatic agent. The hormone might exert its protective effects on hormone-dependent and hormone-independent malignancies predominantly through its MT1 receptor. The *MTNR1A* polymorphisms might play important role for the development and clinical progression of different solid tumors and additionally, they could promote carcinogenic effects of some exogenous factors. The influence of *MTNR1B* variants on carcinogenesis is currently unclear.

Despite the well-known associations between melatonin signaling and reproductive function, the role of melatonin receptor polymorphisms in different reproductive diseases is still not clarified. Additionally, the interrelations between melatonin and melatonin receptor variants are poorly investigated in organ-specific and systemic autoimmune diseases.

Further studies focused on the role of melatonin receptor polymorphisms in different metabolic, reproductive, autoimmune, and oncological diseases might help to identify individuals at risk who might benefit from melatonin administration, lifestyle, and work-schedule changes. Future efforts are needed to uncover the regulation of GPCR melatonin receptors in particular cell context to shed light on their role in health and in the pathogenesis of diseases. The evaluation of melatonin signaling in the context of individual genetic *MTNR1A* and *MTNR1B* haplotypes would be an important step toward personalized medicine.

## Figures and Tables

**Table 1:** Distribution and function of MT1 and MT2 in the immune system *.

Receptor Type	Distribution	Function	Signaling Pathway	Reference
**MT1**	Human peripheral blood mononuclear cells	Regulation of interleukin-2 production Cytoskeletal actin rearrangement CD25+ expression Regulation of interleukin-2 production	Inhibition of cAMP accumulation Inhibition of T-mitogen dependent proliferation Counteraction of prostaglandin EP3 receptor induced Ca^2+^ influx	[46,47,105]
	Jurkat cells	Regulation of interleukin-2 production		[106]
	Human T cells	Regulation of interleukin-2 and interleukin-2 receptor (CD25) production	Inhibition of forskolin-stimulated cyclic AMP (cAMP) production, cyclic GMP (cGMP) production and diacylglycerol (DAG) production	[107,108]
	Human monocytes			[109]
	Mouse peritoneal macrophages		Pertussis toxin-sensitive G protein inhibition of adenylyl cyclase	[110]
	Rat B cells and T cells			[111]
	Rat spleen and thymus	Maturation of secondary immune response		[20,61]
	Mouse splenocytes	Increase of proliferation		[112]
**MT2**	Rat leukocytes	Inhibition of motility (rolling)		[12]
	Chick spleen	Regulation of splenocyte proliferation		[113]
	Jurkat cells	Regulation of of interleukin-2 production		[62]
	Human T cells	Regulation of interleukin-2 and interleukin-2 receptor (CD25) production	cAMP—dependent signaling	[108]
	Human peripheral blood mononuclear cells	Modulation of cell life/death balance of human leucocytes	Inhibition of TNF-α Activation of ERK signaling	[114]
**MT1/MT2**	U937 cells	Prevention of apoptosis induced by ultraviolet irradiation	Activation of extracellular signal-regulated protein kinase (ERK) and mitogen-activated protein kinase (MAPK) pathway	[115,116,117]

* empty columns indicate lack of information.

**Table 2:** Clinical significance of *MTNR1B*, rs10830963 C>G variant.

Clinical Condition	*MTNR1B*, rs10830963 C>G Effects	References
Glucose abnormalities, Beta-cells function, Insulin resistance, Type 2 diabetes mellitus	Minor G-allele is associated with increased fasting glucose as well as with increased prevalence of impaired fasting glucose in children, adolescents and adults. The effect is more pronounced in younger individuals.	[169,176,177,178,184,185,186,187,188,189,190,191,192,193,194,195,196,197,198,199,200,201,202,203,204,205,206,207,208,209,210]
	G-allele is associated with increased risk of type 2 diabetes mellitus in most but not all ethnic groups, and especially in elderly patients.	[169,177,178,182,184,185,188,190,192,202,208,209,211,212,213,214,215,216,217,218,219]
	G allele is associated with increased pancreatic islet *MTNR1B* expression and decreased beta cell glucose sensitivity.	[169,186,191,195,220,221]
	G-allele is associated with decreased early phase insulin secretion after glucose load.	[169,176,178,203,213,222,223,224]
	G allele is not associated with peripheral insulin sensitivity, but is related to hepatic insulin resistance.	[178,186,190]
	G allele determines a significantly higher risk of transition from normal fasting glucose to impaired fasting glucose than from impaired fasting glucose to T2DM.	[181]
Gestational Diabetes Mellitus	G allele is associated with gestational DM in almost all studies. The expression level of MT2 in placenta is significantly higher in the GDM patients than in controls as well as in G allele carriers in comparison to C allele carriers.	[225,226,227,228,229,230,231,232,233,234,235,236,237,238,239,240,241,242,243]
	G allele determines lower effect of lifestyle intervention on the development of GDM.	[234]
	Maternal G allele is associated with higher offspring birth weight.	[244]
Autoimmune diabetes mellitus	G allele is associated with increased risk of low GAD autoantibodies LADA.	[245]
Obesity and weight loss	G allele is not associated with obesity in adults. The polymorphism might exert gender-specific modulation effect on body weight in children.	[176,205,207,246,247]
	G allele determines lower weight loss and weaker improvement of metabolic parameters on hypocaloric diet.	[248,249,250]
	G allele carriers might benefit from hypocaloric low-fat diet leading to a decreased weight, better fat distribution and pronounced decrease of total and LDL- cholesterol in comparison to high-fat diet.	[251,252]
PCOS	G-allele is not associated with increased prevalence of PCOS according to some studies, but is a risk factor for PCOS development according to other studies and a meta-analysis.	[253,254,255,256,257]
	The variant modulates serum glucose and insulin concentrations during OGTT in women with PCOS. G-allele is associated with increased testosterone levels in one, but not other studies.	[253,255,256]
Adolescent idiopathic scoliosis	No significant associations with adolescent idiopathic scoliosis.	[258,259]
Neuro-psychological disturbances	GG genotype associated with an increased risk of postoperative delirium.	[260]
Oncological and autoimmune diseases	Nominal association with increased prostate cancer risk, not significant after correction for multiple comparisons.	[261]
	Possible association with breast cancer risk in some but not all studies, especially in individuals exposed to night-shift work.	[262,263]
	No association with hepatocellular carcinoma risk.	[264]
	Nominal association with increased pancreatic cancer risk.	[265]
	No association with Graves’ disease.	[266]
	G-allele is associated with increased risk of systemic lupus erythematosus in one population, but not in other.	[267,268]
